# CO_2_-based matrix-independent carbon quantification approach for single microplastic-ICP-MS analysis

**DOI:** 10.1007/s00216-025-05934-9

**Published:** 2025-06-06

**Authors:** Kristina Mervič, Agil Azimzada, Mehmet Emin Bayat, Martin Šala, Björn Meermann

**Affiliations:** 1 Division 1.1 – Inorganic Trace Analysis (ITALab), Federal Institute for Materials Research and Testing (BAM), Richard-Willstätter-Straße 11, Berlin, 12489 Germany; 2 Department of Analytical Chemistry, National Institute of Chemistry, Hajdrihova 19, Ljubljana, SI-1000 Slovenia; 3 Division 1.4 – Process Analytical Technology, Federal Institute for Materials Research and Testing (BAM), Richard-Willstätter-Straße 11, Berlin, 12489 Germany

**Keywords:** ICP-MS, Microplastics, Quantification, Gas calibration, Single particle analysis

## Abstract

This study presents a new CO_2_-based, matrix-independent calibration approach for size determination of microplastic (MP) particles using single particle inductively coupled plasma-mass spectrometry (sp-ICP-MS). By incorporating a CO_2_ gas calibration approach and upon adjusting the flow rates, an accurate size determination of MPs between 2 and 7 μm was enabled. Scanning electron microscopy (SEM) was successfully applied for method validation. The CO_2_ gas calibration curves exhibited excellent linearity and reproducibility, with minimal matrix effects across different sample types and matrices, including river water and saline solutions. This CO_2_-based approach eliminates the limitations of matrix- and size-dependent calibrations, enabling robust and accurate size determinations of MPs. This advancement represents a significant step forward in the analytical capabilities for MPs, paving the way for improved environmental monitoring and assessment of plastic pollution.

**Graphical Abstract:**

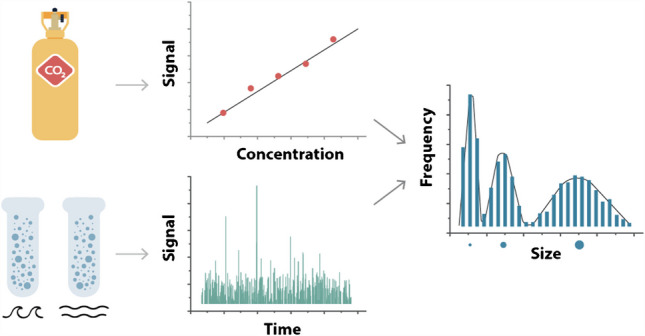

**Supplementary Information:**

The online version contains supplementary material available at 10.1007/s00216-025-05934-9.

## Introduction

In recent decades, microplastics (MPs)—plastic particles between 1 μm and 5 mm [1]—have emerged as a significant threat to the environment and public health. Since their commercial production began in 1950, the world has become increasingly reliant on plastics due to their low production costs, stability, versatility, and light weight [2]. MPs are generated either through the degradation of larger plastic waste or are incorporated into products (microbeads in cosmetics and abrasives, plastic glitter, synthetic fibres from textiles, etc.). The increasing presence of MPs in terrestrial and marine ecosystems has triggered extensive research into their origin, distribution, and potential impacts. In addition, MPs are highly resistant to biodegradation, so once they enter the environment, they can remain there for a long time. This has led to a widespread contamination by MP particles, which have been detected in drinking water and food and pose a potential risk to human health. The bioavailability and toxicity of MPs in the environment are influenced by factors such as their shape, size, surface coating, surface charge, hydrophobicity, and concentration [3]. Therefore, much research has been done to better understand the uptake of MP particles and their subsequent effects on wide range of species of organisms [4–8].

A key priority in addressing the growing concern about MPs is the search for a suitable analytical technique for their accurate detection and quantification, in order to monitor their occurrence, distribution, and movement in the environment [9]. However, the irregular shapes, polymeric varieties, and microscopic sizes of MPs can make their determination a difficult task [10]. Several analytical techniques are currently employed to identify and characterize MPs in the environment, including (i) optical microscopy (polarizing microscopy, scanning electron microscopy (SEM), atomic force microscopy (AFM)), (ii) fluorescence staining [11], (iii) Fourier transform infrared spectrometry [12], (iv) Raman spectrometry [13], (v) laser direct infrared (LDIR) spectrometry [14, 15], (vi) pyrolysis-gas chromatography tandem mass spectrometry (Pyr-GC/MS) [16, 17], (vii) depolymerization-liquid chromatography tandem mass spectrometry (depolymerization-LC/MS) [18], (viii) time-of-flight secondary ion mass spectrometry (ToF-SIMS) [19], and (ix) atmospheric solids analysis probe mass spectrometry (ASAP-MS) [20]. While these techniques are effective in identifying and characterizing MPs, they often come with limitations, such as labor-intensive processes, high costs, and the need for extensive sample preparation. Thus, a combination of different analytical techniques will usually be employed to determine and analyze MPs in diverse environmental matrices.

One of the techniques used for the detection, sizing, and quantification of MP and nanoplastic (NP) particles is single particle inductively coupled plasma-mass spectrometry (sp-ICP-MS) [21]. It is a promising technique that offers high sensitivity, high throughput real-time data on particle size and concentration, and the ability to analyze individual particles. When used in a single particle mode, ICP-MS transforms from a trace metal analysis technique into a particle counting method, providing detailed particle-by-particle information. By analyzing dilute particle suspensions at ultra-high signal acquisition rates of down to 10 μs for AuNP [22] and 100 μs for MPs [23], ICP-MS can detect individual particles and correlate the intensity of particle events with element mass and particle size, provided information on composition, density, and shape is available [24, 25]. However, despite its potential, sp-ICP-MS is not as widely used for MPs analysis as one would expect, primarily because carbon, the main element in plastics, is also ubiquitous, leading to a high background signal. To mitigate signal overload, the less abundant ^13^C isotope is typically analyzed instead of the ^12^C isotope, which comprises the highest abundance. Another challenge in MPs analysis is the introduction of relatively large particles into the system. Nevertheless, a handful of sp-ICP-MS methods have been developed to detect MPs in various contexts [26], featuring notable methodological advancements. For example, Gonzalez de Vega et al. evaluated various sp-ICP-MS strategies, such as the use of different gases and tandem MS, to improve the detection and characterization of polystyrene-based MPs. Their study demonstrated that online aerosol dilution and targeting the ^12^C isotope enhanced the robustness and accuracy of MP analysis in seawater and unicellular organisms [27]. Recent studies have also employed sp-ICP-ToF-MS and laser ablation-sp-ICP-MS for MPs analysis [28–30] and used sp-ICP-MS to gain insights into the aging process of polystyrene (PS) particles under UV light [31].

Calibration is a critical step in ensuring accurate particle size and number determinations of MPs using sp-ICP-MS. While MPs size can be empirically determined by calibrating with particle size standards of the same chemical composition, it can also be estimated via calibration with a dissolved standard of the monitored element, accounting for the analyte transport efficiency (TE) and sample flow rate. Laborda et al. applied both methods to determine MPs in consumer products, using commercially available polystyrene (PS) MP suspensions and aqueous carbon solutions prepared from tartaric acid [26]. In another study, sucrose was employed as a dissolved form of carbon to calibrate PS MPs in the range of 1 to 6 μm [32]. However, one of the key challenges in achieving accurate quantification with sp-ICP-MS is overcoming matrix effects. To address this, it is crucial to develop robust calibration methods and establish validation measures, such as ensuring the availability of reference MPs from diverse sources, before ICP-MS in single-event mode can become a routine method for studying MPs in various samples. Indeed, new calibration approaches are already being explored to improve the reliability and efficiency of sp-ICP-MS measurements. For instance, Harycki et al. utilized online microdroplet calibration, which proved to be a useful approach for the measurement of MPs by correcting for extreme matrix effects [33].

Our study aims to develop and validate an innovative gas calibration technique that offers (i) matrix-independent calibration and (ii) high reproducibility for MPs analysis. This procedure is designed for application in both wet (liquid sample) and dry (e.g., laser ablation of solid samples) aerosol systems, enabling accurate single particle mode analysis via ICP-MS. The method specifically seeks to improve the accuracy of MPs particle size/number determinations in complex natural matrices, thereby enabling more reliable monitoring and assessment of MPs pollution across various environmental media.

## Materials and methods

### Standards and samples

MPs standards used consisted of PS polymers in sizes ranging from 1 to 10 μm. Carboxylated PS MPs in sizes 1.0 μm, 2.0 μm, 3.7 μm (3.5–3.9 μm), and 7.0 μm 5% (w/v) suspensions were supplied by Kisker (Biotech GmbH & Co. KG, Steinfurt, Germany), while 5.28 μm carboxyl PS standard (5% (w/v)) was supplied from Spherotech, Inc. (Lake Forest, IL, USA), 2.86 μm PS standard (10% (w/v)) from MPs, GmbH (Berlin, Germany), and 1.8 μm PS particle standard from BAM (Federal Institute for Materials Research and Testing, Berlin, Germany). A Milli-Q Element water purification system (Merck Millipore) was used to produce ultrapure water (resistivity > 18.2 MΩ cm). The collision reaction cell (CRC) of the ICP-MS was pressurized with pure He (0.5 mL min^−1^). The gas calibration was performed using a single-phase primary reference gas cylinder produced by BAM (cylinder number: 4038–231219) with a composition of 0.99 ± 0.003 cmol/mol CO_2_ (expanded uncertainty *k*=2) and 99.00 ± 0.001 cmol/mol Ar (*k*=2). Additional Ar gas, purchased from Linde GmbH in quality grade 5.0, was used for the dynamic dilution of the reference gas.

### Sample preparation

All MPs suspensions were diluted in Milli-Q water to achieve the appropriate particle number concentrations for plasma introduction, ensuring no overlap in particle-specific (spike) signals. The particle counting statistic was improved via increasing the total acquisition times. River water samples (RW S1 and S2) were collected from two different collection points on the Alte Oder River and filtered with a 0.45-μm polypropylene filter (Cytiva, Whatman Puradisc 25) (pre-rinsed with 10 mL Milli-Q water and 5 mL of river sample). To simulate a seawater matrix, NaCl (TraceSELECT™, Fluka Analytical, Germany) was dissolved in Milli-Q water to prepare solutions with concentrations of 0.001 M, 0.01 M, and 0.1 M. For SEM analysis, 100 μL of MPs particle standards was deposited onto the aluminum carrier and allowed to dry completely for 48 h. To ensure good conductivity and prevent charging during electron bombardment in high vacuum mode, the samples were coated with approximately 30 nm of gold using the Leica EM ACE600.

### Instrumentation and analysis

#### ICP-MS

All measurements for single particle analysis of MPs were carried out using an Agilent 8900 ICP-MS instrument (Agilent Technologies, Japan), equipped with a High Efficiency Sample Introduction System (HE-SIS) and a drainless spray chamber both from Glass Expansion (MA, USA). This type of sample introduction system was used to enhance the signal-to-noise (S/N) ratio for MPs analysis and to accommodate efficient gas/matrix mixing without generating waste. The drainless spray chamber also ensured that all introduced CO_2_ calibration gas was efficiently directed into the plasma. The ICP-MS instrument was operated in a triple quadrupole (QQQ) mode, with both Q1 and Q3 set to an *m*/*z* of 13, and data acquisition was performed with a dwell time of 100 μs. This short dwell time was selected to improve the S/N for MPs by lowering the C background. The acquisition time for a single replicate analysis was 3 min, with five replicates for each setting/sample. To smoothen out C signal fluctuations from the Ar source and extend the linear dynamic range for larger MPs, the ^13^C^+^ signal intensity was slightly suppressed by pressurizing the CRC with a low flow of He (0.5 mL min^−1^). The instrument settings and data acquisition parameters are listed in Table 1. The settings were fine-tuned to achieve high S/N for ^13^C^+^.

**Table 1:** Instrument settings and data acquisition conditions for sp-ICP-MS analysis of MPs suspended in various matrices

Agilent 8900 ICP-MS	
Collision gas	He
Scan type	Triple quad (QQQ)
RF power (W)	1550
Sampling depth (mm)	6.0
CRC gas flow rate (mL min^−1^)	0.5
Extract 1 setting (V)	−9.5
Extract 2 setting (V)	−180
Acquired *m*/*z* ratio (amu)	13
Integration time (μs)	100
Q1 *m*/*z*	13
Q3 *m*/*z*	13
He flow (mL min^−1^)	0.5
Dwell time (μs)	100

#### Introduction system

**Fig. 1:**
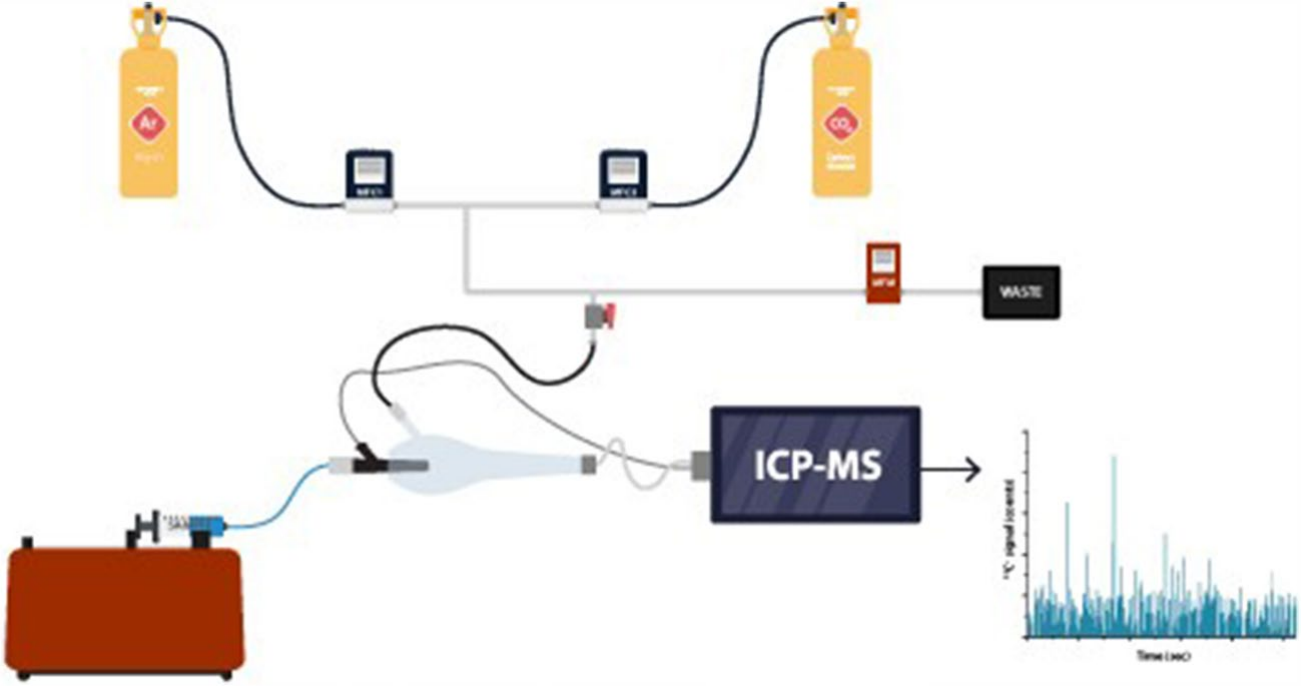
System setup for online gas calibration of MPs size with sp-ICP-MS. The system comprises an argon (Ar) gas source and a carbon dioxide (CO₂) in Ar gas mixture, with their respective flow rates controlled by two bypass thermal mass flow controllers (MFC1 and MFC2). These gas flows are combined before they are introduced into the spray chamber of the ICP-QQQ-MS. To ensure accurate flow control, only a part of the mixed gas was directed into the spray chamber, controlled by an additional valve and monitored by a mass flow meter (MFM) installed on the exhaust line. The remaining 80% of the mixed gas was discharged via the exhaust line (labelled as WASTE). Samples were introduced using a syringe pump equipped with a 2-mL polypropylene syringe to deliver the samples into the drainless spray chamber

The whole introduction system setup is portrayed in Fig. 1. Samples were introduced via a syringe pump (Landgraf Laborsysteme HLL GmbH, Model: LA-100) with a 2-mL polypropylene syringe from Labsolute (without rubber seal), which was calibrated with Milli-Q water gravimetrically beforehand. The sample flow rate was set to 8 μL min^−1^ to enable nebulization with no liquid waste (i.e., total consumption). A drainless spray chamber was used to allow the introduction of CO_2_ for gas calibration. Two bypass thermal mass flow controllers (MFCs) were employed: one for controlling the CO_2_ in Ar gas mixture (MFC model: F-201 CV-050), with a maximum capacity of 50 mLn min^−1^ in argon, and the other for dynamic dilution with argon (MFC model: F-201 CV-500), with a capacity of 500 mLn min^−1^ in argon. Both MFCs, along with their local digital displays and control modules (BRIGHT B2 IP40 9p SubD), were obtained from Bronkhorst Mättig GmbH (Kamen, Germany). The generated flow rates by the MFCs in argon 6.0 (purchased from Linde GmbH) were validated and adjusted. For this purpose, the MFCs were characterized in upstream with laminar flow elements (LFEs) Molbloc L 5E2 and Molbloc L 5E1, purchased from Europascal GmbH (Hanau, Germany). The Molbloc L 5E1, with a maximum quantifiable flow rate of 100 sccm in argon, was used for the MFC F 201 CV-050, while the Molbloc L 5E2, with a maximum quantifiable flow rate of 1000 sccm in argon, was used for the MFC F-201 CV-500. The expanded measurement uncertainty (*k*=2) of the LFEs, which had premium calibration from Fluke Calibration (in compliance with ISO/IEC 17025:2017), was ± 0.125% of the readings. Data from the Molbloc L devices were collected through an in-house GUI developed in Python 3.12.0. The partial gas flows generated by the MFCs (CO_2_ in Ar + Ar) were mixed before being introduced into the spray chamber of the ICP-QQQ-MS system, maintaining a constant total flow rate of 100 mLn min^−1^. Only 20% of the mixed test gas was directed into the ICP-QQQ-MS spray chamber, regulated by an additional valve, and monitored by a mass flow meter (MFM, Analyt-MTC 35807 3464, from Messtechnik GmbH & CO KG) placed on the exhaust line. The MFM was adjusted using the pre-adjusted MFCs, while the remaining 80% of the mixed gas flow was discarded through the exhaust line. For calibration, partial gas flows were set to achieve volume fractions of the CO_2_ in Ar reference material of 5 Vol %, 20 Vol %, 40 Vol %, 60 Vol %, 80 Vol %, and 100 Vol %. This allowed the establishment of a six-point calibration curve with varying amounts of CO_2_ in argon. Due to the capacity limitations of MFC F 201 CV-050, the CO_2_ reference gas was introduced via the low-capacity MFC (F 201 CV-050) for mixing fractions of 5 Vol %, 20 Vol %, and 40 Vol %, while pure argon was introduced via the high-capacity MFC (F 201 CV 500), and vice versa for the mixing fractions 60 Vol %, 80 Vol %, and 100 Vol %. Simultaneously, Milli-Q water was introduced via the HE-SIS nebulizer to ensure matrix-matching with the sample matrix.

#### SEM

SEM characterization of the MPs was conducted using an XL 30 ESEM equipped with a tungsten cathode (FEI, Eindhoven, in 2020 electronic upgrade by point electronic GmbH). The investigations were performed in “high-vacuum mode” of the microscope at an accelerating voltage of 20 keV. Surface topography was imaged using a secondary electron detector (SE detector).

### Data processing

Agilent’s Single Particle Analysis (SPA) software for ICP-MS MassHunter [34] was first employed to generate particle data. The SPA software effectively differentiates single particle signals from background noise by analyzing the characteristics of the ionic (noise) component of the signal distribution using exponential function fitting. This process enables the determination of the intersection between the ionic and particle components of the signal distribution, thereby establishing the threshold for particle detection. Following the separation of particles from the background signal, the intensity of each detected spike (integrated counts per spike event) is proportional to the particle’s mass. Assuming a spherical shape, particle size is then determined from the particle’s mass by comparing the measured intensity to a calibration curve generated from standards of known size and composition. A particle size distribution can then be constructed by binning individual particle events by size and plotting the resulting histogram—a process that was performed using OriginLab. The SEM data were processed using the ImageJ segmentation plugin, where the image was scaled, and particles were segmented and analyzed. The average particle size was determined based on 100 particles. sp-ICP-MS with online gas calibration with CO_2_ was used to analyze the size of PS MPs samples. The diameters of the spherical MPs were calculated based on a density of the PS MPs of 1.05 g cm^−3^ and a mass fraction of carbon in the PS microplastic of 0.9231 [26] (see Electronic Supplementary Material Eq. S1). Using an online gas calibration with CO_2_, the TE factor was calculated in advance using the particle size method [35]. This TE factor was then applied as a correction factor to derive accurate particle size values.

## Results and discussion

### Method development and optimization

#### Sample introduction

The aim of this study was to develop a robust CO_2_-based matrix-independent calibration method to determine the size of MP particles using single particle ICP-MS analysis. Method development and optimization are pivotal in achieving accurate and reliable results; thus, we firstly focused on optimizing the sample introduction system parameters. First, we incorporated an extension line (Tygon tubing, ID: 3.18 mm with a length of about 8 cm), showcased in Fig. 1, between the spray chamber and the plasma to ensure efficient mixing and drying of the aerosols before they reach the plasma. The introduction system, featuring low flow and total consumption configurations [36], ensured no waste and reduced the impact of the matrix—effectively lowering the carbon background without compromising the particle-specific signal and therefore making the method more robust. Next, we determined the maximum CO_2_ flow rate that could be introduced without significantly affecting sensitivity or oxide ratios. After optimization, a total flow rate of 100 mL min^−1^ was used, with 20% of the gas flow directed into the plasma. This configuration successfully covered the dynamic range required for analyzing particles in a size range from 1 to 10 μm without affecting sensitivity or oxide ratios. Lastly, to optimize the S/N, the CRC was pressurized with 0.5 mL min^−1^ He as a collision gas. This adjustment improved the S/N for the detection of MPs by suppressing the background noise originating from the dissolved carbon without necessarily affecting the particle-specific spike signals.

### Gas calibration

**Fig. 2:**
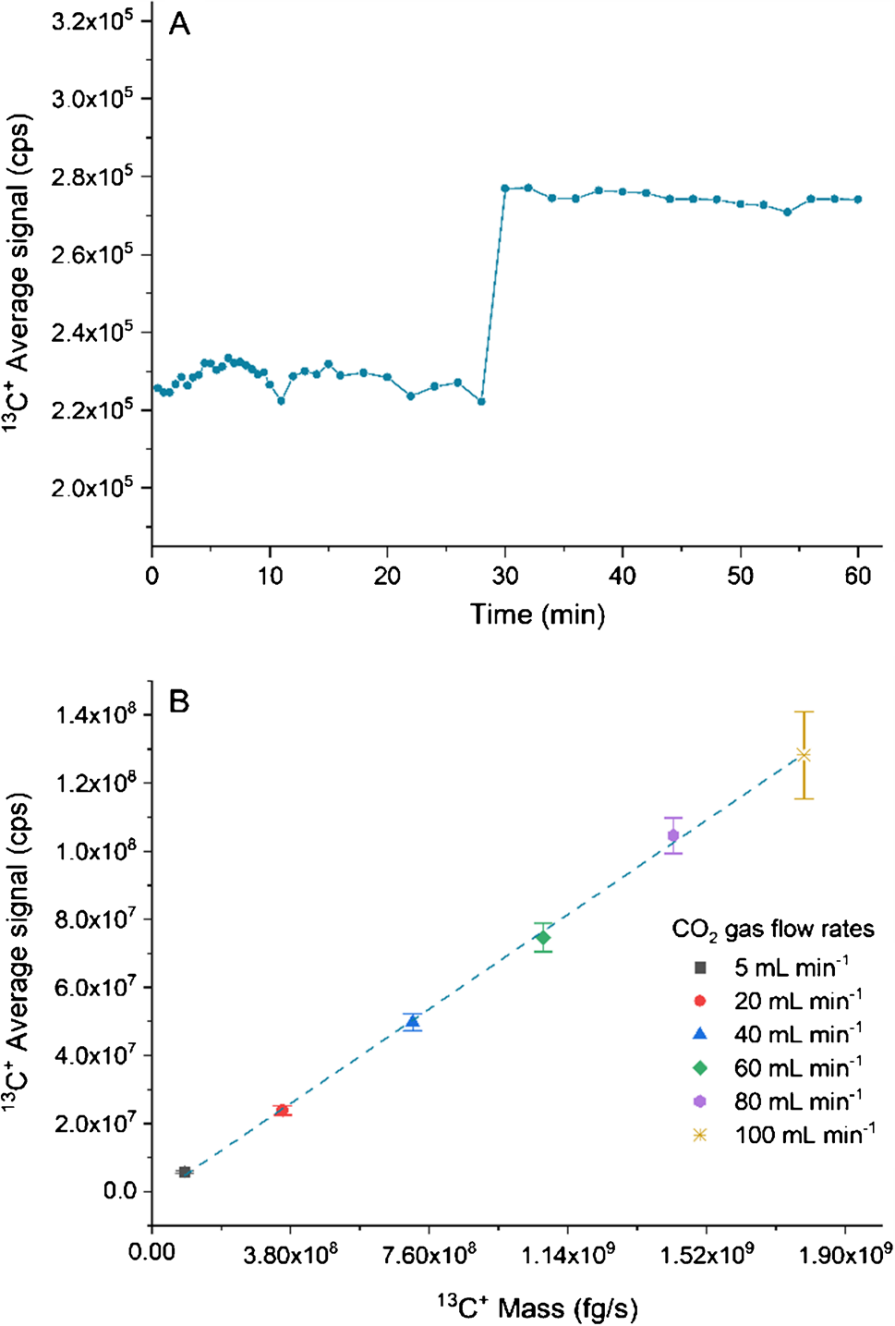
**A** Average ^13^C^+^ signal intensity monitored from the start of online introduction of CO_2_ to assess equilibration time with low flow (2 mL min^−1^) introduced. **B** Average ^13^C^+^ signal intensity as a function of ^13^C^+^ mass introduced via online CO_2_ calibration and analyzed under sp-ICP-MS conditions. Each calibration point represents the average of five replicate measurements

To create the ^13^C^+^ calibration curve, we adjusted the CO_2_ gas flow by varying the ratio of CO_2_ to Ar using the mass flow controllers, vide supra. Depending on the gas mixture, a purge time of 15 to 40 min was required to ensure that the MFC reached thermal equilibrium, thereby providing precise and stable flow rates, with RSDs below 3%. Lower CO_2_ ratios required longer equilibration times. Once the flow was stable (see Fig. 2A), we performed five measurements for each condition to track the intensity of the ^13^C^+^ signals. Throughout these measurements, Milli-Q water (i.e., sample matrix) was continuously added to maintain a consistent matrix, ensuring matrix-matched calibration for the subsequent analyses of MP particles in the Milli-Q. The average ^13^C^+^ signal intensity, plotted against the mass of ^13^C^+^ introduced (calculated from the introduced volumes using the ideal gas law equation, see Electronic Supplementary Material Eq. S2), exhibited a strong linear correlation (*R*^2^ = 0.999, see Fig. 2B).

The setup offers several key advantages: nebulization-independent calibration, matrix-matched calibration, and applicability to dry aerosol measurement systems, such as lasers. The developed method demonstrated a high degree of linearity across the dynamic range (Fig. 2B) and improved relative standard deviations (RSDs), indicating the method’s robustness and reliability.

### Quantification and validation

One of the potential challenges related to MPs analysis via sp-ICP-MS is the possible lack of linearity between the integrated ^13^C^+^ signal and the particle volume, which could result from incomplete evaporation, atomization, and ionization of larger MPs in the ICP ion source, as already observed in the context of sp-ICP-MS analysis of AuNPs [37, 38]. Therefore, MPs in a range from 1 to 10 μm were considered. The use of ultra-short dwell times (100 μs) and pressurization of the CRC with 0.5 mL min^−1^ He ensured a linear dynamic range. In addition, when calibrating the system with CO_2_ gas, a certain amount of gas flows into the spray chamber, vide supra. Thus, to ensure that the conditions remained consistent when switching from CO_2_ gas calibration to particle measurement, argon was introduced as a “make-up” gas. This meant that argon was added to match the total gas flow that was present during the CO_2_ calibration. By doing this, the same overall gas flow into the spray chamber was maintained, which helped ensure that the experimental conditions were identical between the calibration and the actual measurements. This consistency is important for accurate and reliable data, as it minimized variations that could affect the results. Each MPs standard was diluted accordingly for preliminary measurements to obtain a statistically significant particle number concentration for the measurements (see “Materials and methods” section).

**Fig. 3:**
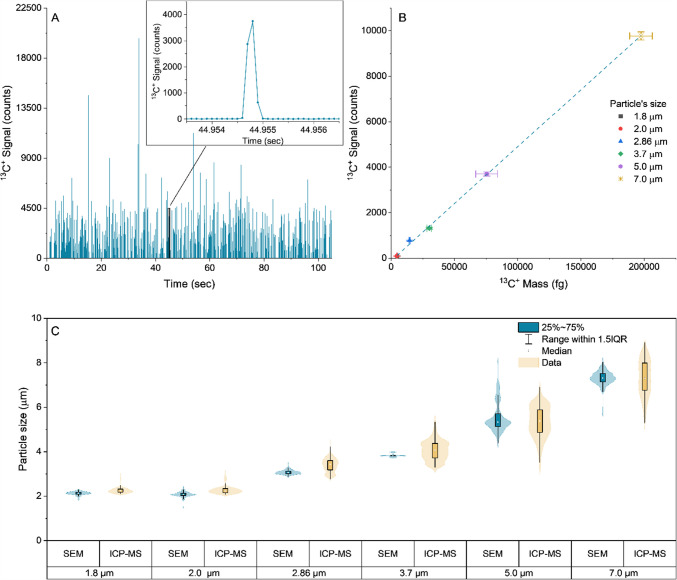
**A** Transient signal observed for 5.3 μm PS MPs. **B** Relationship between the average ^13^C^+^ signal intensity and PS MPs mass, demonstrating high linearity (*R*^2^ > 0.99). **C** Comparison of particle size measurements for PS MPs standards using SEM (based on ~200 particles) and single particle inductively coupled plasma-mass spectrometry (sp-ICP-MS) with CO₂ gas calibration (based on ~1500–3000 particles, depending on the particle number concentration in the sample). The PS MPs standards include particles of sizes 1.8 μm, 2.0 μm, 2.86 μm, 3.7 μm, 5.0 μm, and 7.0 μm

After separating the particles from the background signal, the intensity of each particle event (counts per dwell time interval) was used to determine particle mass and—assuming a spherical shape—also the size. A particle size distribution was then generated by categorizing the individual particle events by size and plotting the resulting histogram to obtain the average signal intensity for each particle size (Fig. 3A). The average ^13^C^+^ signal intensity was then plotted against particle mass, revealing a highly linear correlation (*R*^2^ = 0.999) for particle sizes between 2 and 7 μm (Fig. 3B). This result indicates that PS MPs of at least 7 μm in size are volatilized and atomized in the plasma with consistent efficiency, enabling unbiased sizing of MPs.

SEM was employed to validate the size of our PS MP samples. SEM provides high-resolution images (see Electronic Supplementary Material Fig. S1) that allow for precise measurements of individual particle dimensions, offering a robust method to cross-verify the particle sizes obtained from sp-ICP-MS. The size distribution results from SEM analysis showed a strong correlation with those acquired through sp-ICP-MS with CO_2_ gas calibration, as depicted in Fig. 3C. Specifically, the particle size distribution curves from both SEM and sp-ICP-MS demonstrated similar patterns, confirming the reliability of the sp-ICP-MS measurements. In addition, Fig. 3C shows an overlap between the 1.8 and 2.0 µm MPs. This arises from the characteristics of the standards used. They were sourced from two different manufacturers and labelled as 1.8 µm and 2.0 µm. Both the SEM and ICP-MS analyses consistently showed that the actual average particle size for both standards was approximately 2.0 µm. This explains the overlapping size intervals and indicates that the two standards cannot be distinctly separated by size. This consistency between the two methods highlights the accuracy of our sp-ICP-MS approach in determining the size distribution of PS MPs.

**Fig. 4:**
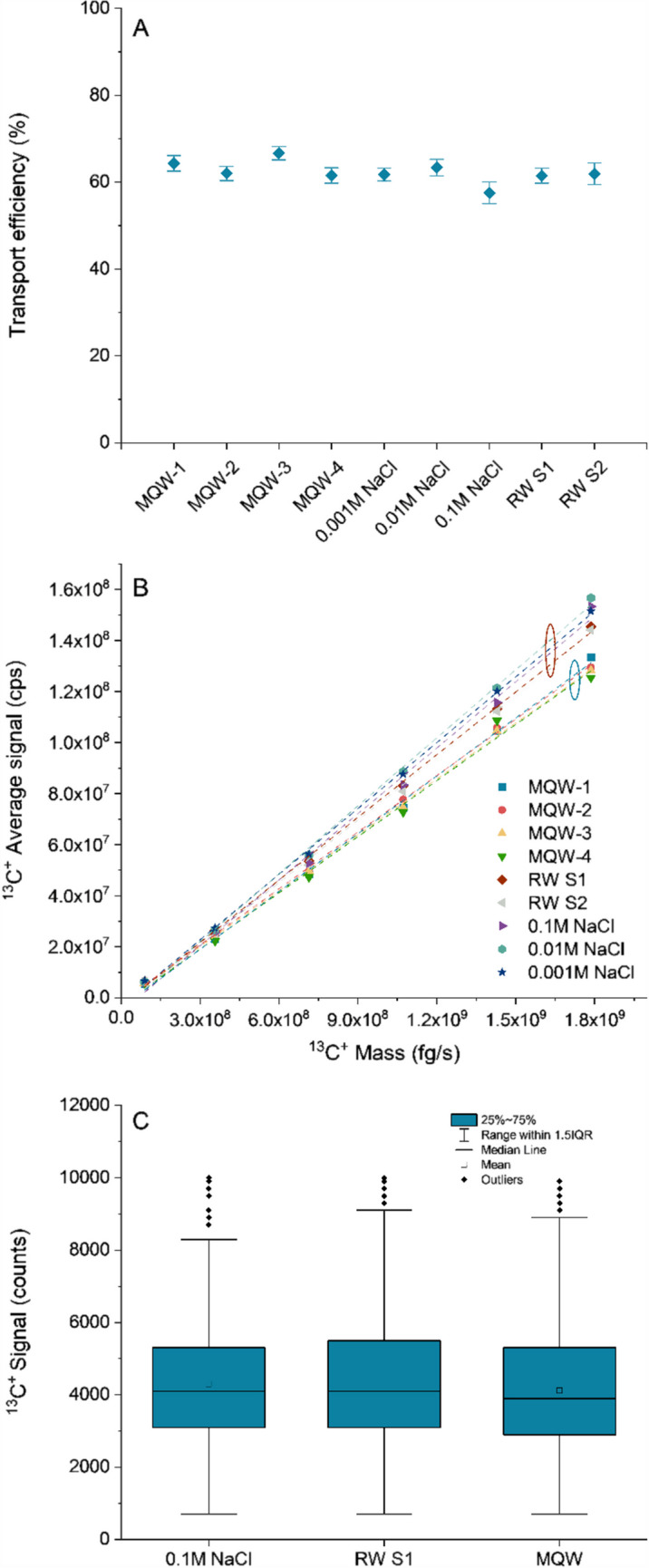
**A** TE for various replicates, including experiments conducted with Milli-Q water on different days, 0.1, 0.01, and 0.001 M NaCl solutions, as well as river water samples (RW S1 and S2) as backgrounds. **B** Average ^13^C^+^ signal intensity as a function of ^13^C^+^ mass introduced via online CO_2_ addition for various replicates, including experiments conducted with Milli-Q water on different days, 0.1, 0.01, and 0.001 M NaCl solutions, as well as river water samples (RW S1 and S2) as backgrounds. **C** ^13^C^+^ integrated signal intensity distributions for 5 μm PS particles analyzed via sp-ICP-MS in various matrices (Milli-Q water, 0.1 M NaCl and river water-RW S74)

In this work, the TE was determined by the particle size method [35] using the 5 μm PS MPs standards. Gold nanoparticles are often used as standards for this method; however, to avoid the differences due to element-specific behavior during transport, ionization, etc., PS particles were used as standards. The method assumes that particles enter the plasma individually (double events are minimized), so that the mass flux into the plasma per single particle event (i.e., integrated counts for the event) corresponds to the mass of a single MP or NP. If the particle size (*d*) and density (*ρ*) are known, the particle mass can be calculated using Eq. S1. If the diameter used in Eq. S1 is the average diameter of a monodisperse particle suspension, then the most frequent pulse intensity (i.e., the peak position of the raw data histogram) should represent the intensity corresponding to the mass of the average size particles. To accurately determine the peak intensity from the binned raw data, a normal distribution was fitted to the histogram using OriginPro 2018. The peak intensity (counts) divided by the particle mass (fg) based on the diameter obtained from the SEM measurements was used as the slope 1. The second step of the particle size method is to create a corresponding dissolved calibration curve. In our case, however, it was a calibration curve for gaseous carbon. The ^13^C^+^ mass was calculated for each calibration point from the volume of CO_2_ entering the sample introduction system according to the ideal gas law Eq. S2. The exact volume was determined using the MFC cells and the MFM cell, which were calibrated with Ar before starting the experiments. The calculated mass was plotted against the average ^13^C^+^ signal intensity, resulting in a six-point calibration curve with the following linear regression equation. The TE of CO₂ calibrant was determined using the response factor of a single particle standard (i.e., 5 μm PS MPs), which is independent of TE and assumed to represent the absolute sensitivity of carbon (i.e., counts per mass-C) in accordance with the particle size method (Pace et al. [35]). In conventional approaches, where both dissolved and particulate samples are introduced through the same inlet (i.e., via liquid nebulization), it is typically assumed that the TE is identical for both phases. In this study, however, the determined TE value pertains solely to CO₂ (critical for particle sizing) and does not represent the TE of the particulate suspension, which was not measured. Notably, although CO₂ introduction constitutes a full-consumption system, TE values below 100% are plausible. This may be attributed, but is not limited, to the diffuse nature of CO₂ as a carbon source, which could result in greater losses at the cone interface, as well as the potential sequestration of CO₂ following the nebulization process (whether from particle suspensions or blanks), thereby reducing its apparent TE. We determined this TE repeatedly for all replicates with Milli-Q water over an extended period to assess the impact of atmospheric changes on the robustness of the method. TE was also calculated for the experiments with 0.1, 0.01, and 0.001 M NaCl solutions (representative for high ionic strength media, e.g., sea water) and river water samples (RW S1 and S2) and is discussed below. The TE was consistently around 65% across all replicates with Milli-Q water, showing minimal variation over time and across different background matrices, including 0.1, 0.01, and 0.001 M NaCl solutions and river water samples (RW S1 and S2) (Fig. 4A). Although we applied a total consumption nebulizer, the TE was 65%, most probably due to the fact that not all of the sample introduced is converted to aerosol, but not all of it reaches the detector; i.e., all of the sample is transported to the plasma where it is ionized, but not all of the ions are efficiently transferred to the MS.

The limit of detection (LOD) was also determined based on the standard deviation of the *y*-axis intercepts of the regression line using Milli-Q water as the matrix. The resulting LOD for the method was determined to be 3077 fg-C per MP; thus, the method can detect down to a particle size of 1.82 μm MPs. This value is in line with those reported in the literature. For example, SP-ICP-MS using direct ^13^C monitoring has been shown to detect polystyrene particles down to 1.2–1.25 µm [26, 39], depending on matrix complexity and sample pre-treatment. Laser ablation SP-ICP-MS (LA-SP-ICP-MS) approaches have reported slightly higher detection limits, around 2.12 µm [28]. It should be noted that particle size detection limits can vary depending on the composition of the particles, as different polymers or elements yield varying instrument responses due to differences in carbon content, ionization efficiency, and matrix effects. Therefore, detection limits should always be interpreted in the context of the specific material under investigation.

### Application and reproducibility

To evaluate the repeatability of the technique, experiments were conducted four times over a month. The comparison of MQ-water calibration curves over time revealed no statistically significant differences, with all *p*-values exceeding 0.05 (*p* > 0.05), as can also be seen from Fig. 4B (blue ellipse). We used an *F*-test to determine whether the two datasets were significantly different from each other, and this analysis was performed for all the samples in comparison to each other. This consistency indicates that the calibration measurements were stable and reliable, confirming that the experimental setup and instrumentation remained consistent throughout the study. Additionally, to test the applicability of the gas calibration method to real samples, multipoint calibration curves were generated using CO₂ gas standards, while real river water samples and sodium chloride solutions (as simulation of a seawater matrix) were introduced instead of Milli-Q water (Fig. 4B, red ellipse). More complex matrices resulted in steeper calibration curves (Fig. 4B, red ellipse) compared to Milli-Q water, this being likely due to the principle of C enhancement [40], where a high concentration of ions in the plasma can also ionize the analyte via charge transfer, leading to increased ionization efficiency and thus steeper calibration slopes. However, all calibration curves in Fig. 4B show a linear correlation with *R*^2^ > 0.995 or higher. In addition, all matrices (Milli-Q water, river water, and sodium chloride solution) were spiked with the same concentration of 5 μm PS microspheres, and the spiked samples were always analyzed directly after the gas calibration curve measurements. The results show that different background matrices do not significantly (*p* ≥ 0.05) affect the distribution of ^13^C^+^ signals for 5 μm PS microspheres (Fig. 4C). This demonstrates the robustness of the method as it can be used to accurately determine the particle size of MPs even in complex matrices.

## Conclusion

Our study contributes to this field by developing and validating a new gas calibration technique for sp-ICP-MS that improves the accuracy and reliability of MP particle size and count determination. By introducing a CO_2_-based calibration method, we have eliminated the main limitations associated with matrix-dependent calibration and improved the robustness and efficiency of the method. The results show a high degree of linearity and consistency between sp-ICP-MS and SEM measurements and underline the potential of the method for routine monitoring of MPs in different environmental matrices. The main benefits of our new approach are (i) its matrix independence as well as (ii) it is much easier to work with CO_2_ than nano/microparticles and more precise. The flows can be accurately set, but the MP standards will always have spread in size and therefore will affect the accuracy of measurements. Furthermore, using MPs as standards, TE needs to be determined, which is also size dependent.

Efforts should be directed towards optimizing the method to address any limitations identified during initial studies, e.g., expanding the detectable size range and upper size limit. The latter was addressed in a study where downward-pointing ICP coupled to a time-of-flight mass spectrometer (ICP-ToF-MS) was developed to address the limitation of introducing large particles into horizontal ICP-MS configurations. This enabled efficient transport and quantitative analysis of large microdroplets, MP beads, and single cells, with results demonstrating its suitability for precise carbon and phosphorus mass measurements in these materials [41]. Additionally, expanding the application of this technique to a broader spectrum of MPs and various environmental samples is crucial. With a small modification, this approach can in fact be used for any MP and thus makes it very general, avoiding the demand for a wide set of standard material which are very hard (if not impossible) to produce, regarding the vast number of different plastic materials. This would involve adapting the calibration process to different types of MPs, including those with varying sizes, shapes, and polymer compositions, as well as applying it to diverse environmental matrices such as soil, water, and biota. All of the above, in combination of ICP-ToF-MS (i.e., simultaneous measuring the whole periodic system) will enable the better identification of plastics (e.g., Cl (PVC)) or specific sources of plastic via detection of metal impurities.

## Supplementary Information

Below is the link to the electronic supplementary material.

Supplementary file 1 (DOCX 759 KB)

## Data Availability

Data will be made available on request from the authors.

## References

[1] Horton AA, Walton A, Spurgeon DJ, Lahive E, Svendsen C. Microplastics in freshwater and terrestrial environments: evaluating the current understanding to identify the knowledge gaps and future research priorities. Sci Total Environ. 2017;586:127–41. 28169032 10.1016/j.scitotenv.2017.01.190

[2] Hale RC, Seeley ME, La Guardia MJ, Mai L, Zeng EY. A global perspective on microplastics. J Geophys Res Oceans. 2020;125(1):e2018JC014719.

[3] Wright SL, Thompson RC, Galloway TS. The physical impacts of microplastics on marine organisms: a review. Environ Pollut. 2013;178:483–92. 23545014 10.1016/j.envpol.2013.02.031

[4] Vethaak AD, Legler J. Microplastics and human health. Science. 2021;371(6530):672–4. 33574197 10.1126/science.abe5041

[5] Wright SL, Kelly FJ. Plastic and human health: a micro issue? Environ Sci Technol. 2017;51(12):6634–47. 28531345 10.1021/acs.est.7b00423

[6] Zientek A, Schagerl M, Nagy M, Wanek W, Heinz P, Ali SS, et al. Effect of micro-plastic particles on coral reef foraminifera. Sci Rep. 2024;14(1):6634–47. 38816478 10.1038/s41598-024-63208-3 PMC11139942

[7] Gündoğdu S, Bour A, Köşker AR, Walther BA, Napierska D, Mihai F-C, et al. Review of microplastics and chemical risk posed by plastic packaging on the marine environment to inform the Global Plastics Treaty. Sci Total Environ. 2024;946: 174000. 38901589 10.1016/j.scitotenv.2024.174000

[8] Digka N, Patsiou D, Hatzonikolakis Y, Raitsos DE, Skia G, Koutsoubas D, et al. Microplastic ingestion in mussels from the East Mediterranean Sea: exploring its impacts in nature and controlled conditions. Sci Total Environ. 2024;946:174268. 38925375 10.1016/j.scitotenv.2024.174268

[9] Huang Z, Hu B, Wang H. Analytical methods for microplastics in the environment: a review. Environ Chem Lett. 2023;21(1):383–401. 36196263 10.1007/s10311-022-01525-7 PMC9521859

[10] Silva AB, Bastos AS, Justino CIL, da Costa JP, Duarte AC, Rocha-Santos TAP. Microplastics in the environment: challenges in analytical chemistry - a review. Anal Chim Acta. 2018;1017:1–19. 29534790 10.1016/j.aca.2018.02.043

[11] Liu S, Shang E, Liu J, Wang Y, Bolan N, Kirkham MB, et al. What have we known so far for fluorescence staining and quantification of microplastics: a tutorial review. Front Environ Sci Eng. 2021;16(1):8.

[12] Möller JN, Löder MGJ, Laforsch C. Finding microplastics in soils: a review of analytical methods. Environ Sci Technol. 2020;54(4):2078–90. 31999440 10.1021/acs.est.9b04618

[13] Allen S, Allen D, Phoenix VR, Le Roux G, Durántez Jiménez P, Simonneau A, et al. Atmospheric transport and deposition of microplastics in a remote mountain catchment. Nat Geosci. 2019;12(5):339–44.

[14] Cheng Y-L, Zhang R, Tisinger L, Cali S, Yu Z, Chen HY, et al. Characterization of microplastics in sediment using stereomicroscopy and laser direct infrared (LDIR) spectroscopy. Gondwana Res. 2022;108:22–30.

[15] Luo X, Wang Z, Yang L, Gao T, Zhang Y. A review of analytical methods and models used in atmospheric microplastic research. Sci Total Environ. 2022;828:154487. 35278538 10.1016/j.scitotenv.2022.154487

[16] Fischer M, Scholz-Böttcher BM. Simultaneous trace identification and quantification of common types of microplastics in environmental samples by pyrolysis-gas chromatography–mass spectrometry. Environ Sci Technol. 2017;51(9):5052–60. 28391690 10.1021/acs.est.6b06362

[17] Hendrickson E, Minor EC, Schreiner K. Microplastic abundance and composition in Western Lake Superior as determined via microscopy, Pyr-GC/MS, and FTIR. Environ Sci Technol. 2018;52(4):1787–96. 29345465 10.1021/acs.est.7b05829

[18] Liu C, Li J, Zhang Y, Wang L, Deng J, Gao Y, et al. Widespread distribution of PET and PC microplastics in dust in urban China and their estimated human exposure. Environ Int. 2019;128:116–24. 31039519 10.1016/j.envint.2019.04.024

[19] Du C, Wu J, Gong J, Liang H, Li Z. ToF-SIMS characterization of microplastics in soils. Surf Interface Anal. 2020;52(5):293–300.

[20] Vitali C, Janssen H-G, Ruggeri FS, Nielen MWF. Rapid single particle atmospheric solids analysis probe-mass spectrometry for multimodal analysis of microplastics. Anal Chem. 2023;95(2):1395–401. 36547121 10.1021/acs.analchem.2c04345 PMC9850409

[21] Kutralam-Muniasamy G, Shruti VC, Pérez-Guevara F, Flores JA. The emerging field of inductively coupled plasma mass spectrometry for (micro)nanoplastic analysis: “the 3As” analysis, advances, and applications. Trends Analyt Chem. 2024;174:117673.

[22] Shaw P, Donard A. Nano-particle analysis using dwell times between 10 μs and 70 μs with an upper counting limit of greater than 3 × 107 cps and a gold nanoparticle detection limit of less than 10 nm diameter. J Anal At Spectrom. 2016;31(6):1234–42.

[23] Bolea-Fernandez E, Rua-Ibarz A, Velimirovic M, Tirez K, Vanhaecke F. Detection of microplastics using inductively coupled plasma-mass spectrometry (ICP-MS) operated in single-event mode. J Anal At Spectrom. 2020;35(3):455–60.

[24] Montaño MD, Olesik JW, Barber AG, Challis K, Ranville JF. Single particle ICP-MS: advances toward routine analysis of nanomaterials. Anal Bioanal Chem. 2016;408(19):5053–74. 27334719 10.1007/s00216-016-9676-8

[25] Mozhayeva D, Engelhard C. A critical review of single particle inductively coupled plasma mass spectrometry – a step towards an ideal method for nanomaterial characterization. J Anal At Spectrom. 2020;35(9):1740–83.

[26] Laborda F, Trujillo C, Lobinski R. Analysis of microplastics in consumer products by single particle-inductively coupled plasma mass spectrometry using the carbon-13 isotope. Talanta. 2021;221:121486. 33076096 10.1016/j.talanta.2020.121486

[27] Gonzalez de Vega R, Goyen S, Lockwood TE, Doble PA, Camp EF, Clases D. Characterisation of microplastics and unicellular algae in seawater by targeting carbon via single particle and single cell ICP-MS. Anal Chim Acta. 2021;1174, 338737. 10.1016/j.aca.2021.338737 34247735

[28] Van Acker T, Rua-Ibarz A, Vanhaecke F, Bolea-Fernandez E. Laser ablation for nondestructive sampling of microplastics in single-particle ICP-mass spectrometry. Anal Chem. 2023;95(50):18579–86. 38050919 10.1021/acs.analchem.3c04473

[29] Brunnbauer L, Jirku M, Quarles CD, Limbeck A. Capabilities of simultaneous 193 nm - LIBS/LA-ICP-MS imaging for microplastics characterization. Talanta. 2024;269:125500. 38070285 10.1016/j.talanta.2023.125500

[30] Pořízka P, Brunnbauer L, Porkert M, Rozman U, Marolt G, Holub D, et al. Laser-based techniques: novel tools for the identification and characterization of aged microplastics with developed biofilm. Chemosphere. 2023;313:137373. 36435319 10.1016/j.chemosphere.2022.137373

[31] Liu Z, Zhu Y, Lv S, Shi Y, Dong S, Yan D, et al. Quantifying the dynamics of polystyrene microplastics UV-aging process. Environ Sci Technol Lett. 2021;9:50–6.

[32] Sakanupongkul A, Sirisinha K, Saenmuangchin R, Siripinyanond A. Analysis of microplastic particles by using single particle inductively coupled plasma mass spectrometry. Microchem J. 2024;199:110016.

[33] Harycki S, Gundlach-Graham A. Single-particle ICP-TOFMS with online microdroplet calibration: a versatile approach for accurate quantification of nanoparticles, submicron particles, and microplastics in seawater. Anal Chem. 2023;95(41):15318–24. 37788319 10.1021/acs.analchem.3c02785

[34] Takayuki Itagaki SW, Michiko Yamanaka, inventor; Agilent Technologies, Inc. , assignee. Automated detection of nanoparticles using single-particle inductively coupled plasma mass spectrometry (SP-ICP-MS) patent 11075066. 2021.

[35] Pace HE, Rogers NJ, Jarolimek C, Coleman VA, Higgins CP, Ranville JF. Determining transport efficiency for the purpose of counting and sizing nanoparticles via single particle inductively coupled plasma mass spectrometry. Anal Chem. 2011;83(24):9361–9. 22074486 10.1021/ac201952t PMC3410750

[36] Tharaud M, Louvat P, Benedetti MF. Detection of nanoparticles by single-particle ICP-MS with complete transport efficiency through direct nebulization at few-microlitres-per-minute uptake rates. Anal Bioanal Chem. 2021;413(3):923–33. 33236223 10.1007/s00216-020-03048-y

[37] Ho K-S, Lui K-O, Lee K-H, Chan W-T. Considerations of particle vaporization and analyte diffusion in single-particle inductively coupled plasma-mass spectrometry. Spectrochim Acta, Part B. 2013;89:30–9.

[38] Lee W-W, Chan W-T. Calibration of single-particle inductively coupled plasma-mass spectrometry (SP-ICP-MS). J Anal At Spectrom. 2015;30(6):1245–54.

[39] Trujillo C, Pérez-Arantegui J, Lobinski R, Laborda F. Improving the detectability of microplastics in river waters by single particle inductively coupled plasma mass spectrometry. Nanomaterials. 2023;13(10):2582. 37241999 10.3390/nano13101582 PMC10223556

[40] Narukawa T, Iwai T, Chiba K. An ICP index for ICP-MS determinations – new selection rules for internal standards in ICP-MS determinations and carbon enhancement effect. J Anal At Spectrom. 2017;32(8):1547–53.

[41] Vonderach T, Gundlach-Graham A, Günther D. Determination of carbon in microplastics and single cells by total consumption microdroplet ICP-TOFMS. Anal Bioanal Chem. 2024;416(11):2773–81. 38062197 10.1007/s00216-023-05064-0 PMC11009739

