# Supplementary material for: CO2-based matrix-independent carbon quantification approach for single microplastic-ICP-MS analysis
Source: Anal Bioanal Chem. 2025 Jun 6;418(16):5071–81. doi: 10.1007/s00216-025-05934-9 (PMC13424213; doi:10.1007/s00216-025-05934-9)
Supplement: Supplementary file 1 — (DOCX 759 KB) [file 216_2025_5934_MOESM1_ESM.docx]

Electronic supplementary information

**CO_2_-based matrix-independent carbon quantification approach for single microplastic-ICP-MS analysis**

Kristina Mervič ^2‡^, Agil Azimzada^1‡^, Mehmet Emin Bayat^3^, Martin Šala ^2^*, Björn Meermann^1^*

^1^ Federal Institute for Materials Research and Testing (BAM), Division 1.1 – Inorganic Trace Analysis (ITALab), Richard-Willstätter-Straße 11, 12489 Berlin

^2^ National Institute of Chemistry, Department of Analytical Chemistry, Hajdrihova 19, SI-1000 Ljubljana, Slovenia

^3^Federal Institute for Materials Research and Testing (BAM), Division 1.4 – Process Analytical Technology, Richard-Willstätter-Straße 11, 12489 Berlin

*Corresponding authors: Dr. Martin Šala, Email: [Martin.Sala@ki.si](mailto:Martin.Sala@ki.si) & PD Dr. habil. Björn Meermann, Email: [bjoern.meermann@bam.de](mailto:bjoern.meermann@bam.de)

‡Kristina Mervič and Agil Azimzada are equal first authors of this manuscript

**Equations**

$m_{MPs}=\rho\frac{3}{4}\pi\left( \frac{d}{2} \right)^{3}$ (1)

$pV=nRT$ (2)

$TE= \frac{\frac{{counts}_{MPs}}{m_{MPs}}}{\frac{{counts}_{gas}}{m_{gass}}}100$ (3)

**Figures**


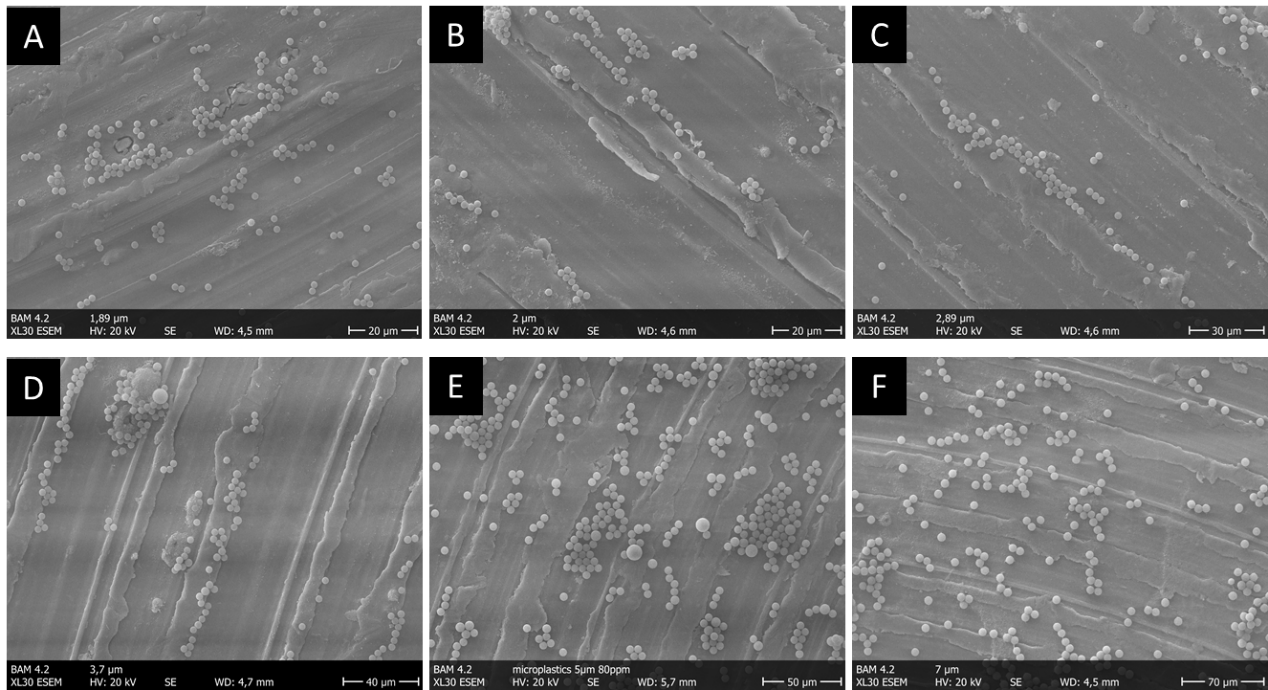


Figure S1 SEM images of 1.8 μm (A), 2.0 μm (B), 2.86 μm (C), 3.7 μm (D), 5.0 μm (E), and 7.0 μm (F) polystyrene microspheres.
